# Will Mobile Diabetes Education Teams (MDETs) in primary care improve patient care processes and health outcomes? Study protocol for a randomized controlled trial

**DOI:** 10.1186/1745-6215-13-165

**Published:** 2012-09-13

**Authors:** Enza Gucciardi, Mariella Fortugno, Stacey Horodezny, Wendy Lou, Souraya Sidani, Sherry Espin, Fiona Webster, Baiju R Shah

**Affiliations:** 1 Ryerson University, School of Nutrition, 350 Victoria Street, Toronto, ON M5B 2 K3, Canada; 2 Trillium Health Centre, 5770 Hurontario Street, Mississauga, ON, L5R 3 G5, Canada; 3 University of Toronto, Dalla Lana School of Public Health, 155 College Street, Health Science Building, 6th floor, Toronto, ON, M5T 3 M7, Canada; 4 Ryerson University, School of Nursing, 350 Victoria Street, Toronto, ON, M5B 2 K3, Canada; 5 Sunnybrook Research Institute, 2075 Bayview Avenue, Toronto, ON, M4N 3 M5, Canada; 6 Institute for Clinical Evaluative Sciences, G1 06, 2075 Bayview Avenue, Toronto, ON, M4N 3 M5, Canada; 7 Department of Medicine, Suite RFE 3-805, University of Toronto, 200 Elizabeth Street, Toronto, ON, M5G 2C4, Canada

**Keywords:** Diabetes, Self-management education, Diabetes self-management support, Primary care, Cluster randomized controlled trial, Stepped wedge design, Inter-professional collaboration, Chronic disease models

## Abstract

**Background:**

There is evidence to suggest that delivery of diabetes self-management support by diabetes educators in primary care may improve patient care processes and patient clinical outcomes; however, the evaluation of such a model in primary care is nonexistent in Canada. This article describes the design for the evaluation of the implementation of Mobile Diabetes Education Teams (MDETs) in primary care settings in Canada.

**Methods/design:**

This study will use a non-blinded, cluster-randomized controlled trial stepped wedge design to evaluate the Mobile Diabetes Education Teams' intervention in improving patient clinical and care process outcomes. A total of 1,200 patient charts at participating primary care sites will be reviewed for data extraction. Eligible patients will be those aged ≥18, who have type 2 diabetes and a hemoglobin A1c (HbA1c) of ≥8%. Clusters (that is, primary care sites) will be randomized to the intervention and control group using a block randomization procedure within practice size as the blocking factor. A stepped wedge design will be used to sequentially roll out the intervention so that all clusters eventually receive the intervention. The time at which each cluster begins the intervention is randomized to one of the four roll out periods (0, 6, 12, and 18 months). Clusters that are randomized into the intervention later will act as the control for those receiving the intervention earlier. The primary outcome measure will be the difference in the proportion of patients who achieve the recommended HbA1c target of ≤7% between intervention and control groups. Qualitative work (in-depth interviews with primary care physicians, MDET educators and patients; and MDET educators’ field notes and debriefing sessions) will be undertaken to assess the implementation process and effectiveness of the MDET intervention.

**Trial registration:**

ClinicalTrials.gov NCT01553266

## Background

Diabetes mellitus is a chronic illness that requires a lifelong commitment to complex lifestyle modifications involving nutrition management, a physically active lifestyle, regular self-monitoring of blood glucose and adherence to medications and/or insulin therapy. Proper management of these lifestyle modifications have been shown to reduce the risk and progression of diabetes complications [1-6]. Achieving and sustaining effective management is challenging; more than half of those living with diabetes in Canada are unable to meet the recommended target for glycemic control (≤7% hemoglobin A1c (HbA1c)) [7]. As a result, diabetes self-management support (DSMS) is recommended by Canadian clinical practice guidelines for the management of diabetes as a valuable resource that supports and teaches patients the skills to actively participate in the management of their illness [8].

DSMS is a collaborative process through which people with, or at risk for, diabetes gain the knowledge and skills needed to modify behavior and successfully self-manage the disease and its related conditions. It is an interactive, ongoing process involving the person with diabetes (and possibly the caregiver and/or family) and a diabetes educator(s), and focuses on essential self-care behaviors, such as healthy eating, being active, monitoring blood glucose, taking medication, problem solving, healthy coping and reducing risks of further complications [9]. DSMS primarily relies on patients and diabetes educators to define management issues, set priorities, establish collaborative goals, identify barriers, create a management plan and problem-solve to maintain optimal management [10,11]. The few studies that exist on the delivery of DSMS in primary care have found that DSMS has numerous benefits on patient clinical and care process outcomes, such as an improvement in patient knowledge [12-14]; a reduction in body weight [13], HbA1c [12-16], cholesterol [14-17], fasting blood glucose [13,18], self-blood glucose monitoring [16] and blood pressure [18]; and improved primary care physician (PCP) adherence to diabetes clinical practice guidelines [14,17]. However, none of these studies have been conducted within a Canadian setting.

Many diabetes education programs (DEPs) across Ontario and Canada are starting to provide outreach services by creating collaborative partnerships with local PCPs, and establishing mobile diabetes education teams (MDETs) that deliver DSMS to patients and provide diabetes training and support to PCPs within primary care sites. Models of care that integrate multidisciplinary teams have been shown to improve patient access to DSMS [19] and increase physician support to reduce clinical inertia in the area of diabetes management in primary care [20-24]. Furthermore, there is growing evidence that inter-professional collaboration (IPC), whereby professionals from different health disciplines collaborate together to achieve a shared goal, such as quality patient care [25], can improve patient care processes and health outcomes [26-42]. Thus, to manage diabetes effectively, alternative models of practice that integrate inter-professional teams may be required to reduce the burden of diabetes.

This protocol is for the evaluation of the implementation of MDETs in primary care in Canada. This model of practice supports PCPs at various primary care sites to assist in and share the care and management of patients with diabetes by offering a MDET (one registered nurse (RN) and one registered dietitian (RD)) onsite, one to four times a month based on patient volume (see Table 1 for an overview of the MDET intervention). The proposed MDET intervention is informed by the five components of the Chronic Care Model (CCM) [19]: (i) Organization of integrative patient health care; (ii) Forming community linkages; (iii) Encouraging self-management support; (iv) Providing decision support; and (v) Delivery system redesign (see Table 2). This article describes the non-blinded, cluster-randomized trial stepped wedge design used in the three-year evaluation study of the MDET intervention in primary care.

**Table 1:** Mobile Diabetes Education Team (MDET) intervention components, timelines and content

**Intervention component**	**Timelines**	**Content**
RN initial visit	Patient’s first visit with RN, one hour	**Assessment:** Time-span with diabetes; diabetes knowledge; recent hospital admissions; medication regime and adherence; lab blood work review; medical history; blood pressure; foot exam; capacity for SBGM and/or results review; current exercise regime; presence of diabetes complications; risk management for new and existing acute and long-term complications; proper identification of diabetes.
		**Education:** What is diabetes?; how medications work; signs and symptoms of high and low blood sugars; how to reduce long-term complications; benefits of exercise; SBGM technique and timing; how to interpret SBGM results; sharps disposal; stress management; foot care.
		**Teach as needed:** Insulin time action; insulin administration and technique; titrating doses; sick day management; travel glucagon and ketones.
RD initial visit	Patient’s first visit with RD, one hour	**Assessment:** Effects of food, exercise and medications on blood glucose levels and cholesterol; lab blood work review; weight history and goals; fast food consumption; meal composition and timing; specialty foods, supplements, and alcohol use; food allergies; disordered eating.
		**Education:** Healthy eating guidelines; meal planning, food choices and portion size; carbohydrate load and consistency; major food nutrients; specialty food and sweeteners; fiber, fats, sodium, and alcohol; eating out.
		**Teach as needed:** Carbohydrate counting; glycemic index; insulin carbohydrate ratio.
Action plan and goal setting	At initial MDET visits, and ongoing	MDET helps patient find behaviors they would like to initiate or change; patient and MDET collaboratively create action plan and set goals. This may include a referral to a local DEP for additional support (for example, cooking demos, and so on). Goals are then shared with the PCP.
Collaborative patient care/Patient case conferencing	Following each patient visit with MDET	MDET communicates with PCP through face-to-face meetings or using a communication tool to review major obstacles to good diabetes control and make recommendations about medication changes to help improve control for the patient. Goals are decided upon by the patient as what they would like to work on before the next visit, and are reinforced by the PCP on following visits.
MDET follow-up visits	30 minutes x 3 visits over 1-year period	**Assessment:** Patients’ success in achieving established goals; future goals; SBGM results and recent blood work to determine diabetes control; effectiveness of medication and lifestyle changes.
		**Education:** Teach topics listed in RN and RD initial visit guidelines, based on identified issues from first initial visit, patient questions and follow-up assessment.

DEP, Diabetes education program; MDET, Mobile Diabetes Education Team; PCP, Primary care physician; RD, Registered dietician; RN, Registered nurse; SBGM, Self blood glucose monitoring.

**Table 2:** Chronic Care Model – conceptual framework for MDETs in primary care

**CCM component**	**Operationalization of CCM components**	**Research objective(s)**
Organized integrated patient care	· Diabetes self-management support is being offered in PCPs’ offices for patients with diabetes.	i. Examine the effect of MDETs on patient clinical outcomes.
	· An opportunity is created to promote knowledge exchange and capacity between PCPs and MDETs/DEPs by working together to provide better care for patients in primary care.	ii. Evaluate the effect of MDETs on the quality of care delivered to patients by PCPs.
		iii. Examine the effect of MDETs on physician referral and patient utilization of DEPs.
		iv. Assess the implementation process of the MDET and the degree of IPC between PCPs and MDETs across primary care sites.
Community linkages	· An opportunity is created to promote knowledge exchange and capacity between PCPs and MDETs/DEPs by working together to provide better care for patients in primary care.	iii. Examine the effect of MDETs on physician referral and patient utilization of DEPs.
		iv. Assess the implementation process of the MDET and the degree of IPC between PCPs and MDETs across primary care sites.
Patient self-management support	· Diabetes self-management support is being offered in PCPs’ offices for patients with diabetes.	i. Examine the effect of MDETs on patient clinical outcomes.
Provider decision support	· An opportunity is created to promote knowledge exchange and capacity between PCPs and MDETs by working together to provide better care for patients in primary care.	i. Examine the effect of MDETs on patient clinical outcomes.
	· A communication tool will be used to exchange patient recommendations and treatment plans between MDETs and PCPs.	ii. Evaluate the effect of MDETs on the quality of care delivered to patients by PCPs.
	· Regular case conferences have been agreed upon between MDETs and PCPs after patients’ visits.	iii. Examine the effect of MDETs on physician referral and patient utilization of DEPs.
	· Hard copy of clinical practice guidelines and Diabetes Flow Sheet for Diabetes Management for PCPs.	iv. Assess the implementation process of the MDET and the degree of IPC between PCPs and MDETs across primary care sites
Delivery system re-design	· Diabetes self-management support is being offered in PCPs’ offices for patients with diabetes.	i. Examine the effect of MDETs on patient clinical outcomes.
	· An opportunity is created to promote knowledge exchange and capacity between PCPs and MDETs/DEPs by working together to provide better care for patients in primary care.	ii. Evaluate the effect of MDETs on the quality of care delivered to patients by PCPs.
		iii. Examine the effect of MDETs on physician referral and patient utilization of DEPs.
		iv. Assess the implementation process of the MDET and the degree of IPC between PCPs and MDETs across primary care sites.

DEP, Diabetes education program; IPC, Inter-professional collaboration; MDET, Mobile Diabetes Education Team; PCP, Primary care physician.

### Aims of the study

The objectives of this study (see Table 2) are to evaluate the effect of MDETs on: 1)patient clinical outcomes; 2) the quality of care delivered to patients; and 3) PCPs’ referrals to and patients’ utilization of DEPs. We will also be examining the implementation process of the MDETs and the degree of IPC that occurs between PCPs and MDETs across primary care sites.

### Scientific hypothesis

We hypothesize that the MDET intervention results in the following outcomes at 12 months:

(i) An improvement in patient clinical outcomes, primarily an increase, or greater proportion, of patients in the intervention group who are at target for HbA1c (≤7%) compared to patients in the control group.(Secondary hypothesis: an increase, or greater proportion, of patients in the intervention group who are at target for low-density lipoprotein (LDL-C) ≤2.0 mmol/L, total cholesterol-high density lipoprotein ratio (TC-HDL ratio) < 4.0, and blood pressure ≤ 130/80 mmHg, compared to patients in the control group).

(ii) An increase in the proportion of PCPs performing patient care processes, according to the Canadian clinical practice guidelines for the management of diabetes, in the intervention group compared to the control group.

(iii) An increase in the proportion of referrals to and patients’ utilization of DEPs in the intervention group compared to one year prior to the start of the intervention.

## Methods/design

### Setting

The MDETs in primary care sites, including independent practices, family health teams, walk-in clinics and community healthcare centers, will take place in several suburban cities and towns in Ontario. The region’s population is one of the largest with more than one million residents.

### Participants

The study involves two groups of participants: PCPs and patients assigned to their care. PCPs will be selected if they agree to refer patients with type 2 diabetes to the MDET. Patients will be included if they are ≥18 years of age, have type 2 diabetes and have an HbA1c of ≥8%. Patients who are newly diagnosed or have lived with the disease for many years are eligible because the primary treatment/management goal is to achieve glycemic control and other clinical targets to reduce the risk of complications. As such, we plan to include both prevalent and incident cases, but restrict inclusion only to patients who are at high risk (≥8%) in our research sample. Patients who have a type of diabetes that requires intense and specialized treatment, such as type 1 diabetes, gestational diabetes or patients on a complex multiple daily insulin regime, will be excluded. Ethical approval for this study has been granted by the Ryerson University Research Ethics Board, including the waiver of consent to review patient medical charts. Informed consent for all other data collection activities will be gained from all participants.

### Experimental study design

A non-blinded, cluster-randomized trial stepped wedge design will be used (see Table 3). Findings from such a study design have been shown to generate sound scientific evidence and may better facilitate the implementation of complex health interventions in practice [43]. We propose to randomize clusters (that is, primary care sites) and not individuals to the intervention and control group in order to minimize contamination or treatment dissemination between study groups (intervention or control). The presence of a MDET is hypothesized to affect how PCPs care for their patients; and thus, the care patients receive within the same practice site.

**Table 3:** Study design – cluster randomized control trial stepped wedge design treatment schedules

**Randomization of clusters**	**Time 1**	**Time 2**	**Time 3**	**Time 4**	**Time 5**
	0 months*	**6 months**	**12 months**	**18 months**	**24 months**
Cluster 1	1	1	1	1	1
Cluster 2	0	1	1	1	1
Cluster 3	0	0	1	1	1
Cluster 4	0	0	0	1	1

“*” represents baseline data collection across all sites prior to cluster 1 starting the intervention;

“0” represents control or existing treatment/standard care at primary care sites; “1” represents the MDET intervention; each column represents a data collection point.

Because all PCPs who expressed an interest in this study are eager to have a MDET on site, we plan to use a stepped wedge design to sequentially roll out the intervention. The time at which each cluster begins the intervention is randomized to one of the four roll-out periods (0, 6, 12, and 18 months). The randomization times are separated by six months to allow for assessment of the primary outcome (that is, HbA1c) within a reasonable time after implementation of the intervention [13]. Clusters that are randomized into the intervention later will act as the control for the clusters who receive the intervention earlier. All clusters will subsequently receive the intervention, providing additional evidence of the intervention’s effectiveness. Using computer-generated random numbers, the team statistician blinded to the identity of the sites will randomize sites using a block randomization procedure within practice size (determined by the number of PCPs practicing at each site) as the blocking factor. Blocking on practice size will ensure that this site characteristic will be equally distributed across study groups. For the clusters receiving the intervention in the first year, 6 and 12 months of follow-up data collection have been built into the study design (18 and 24 months) to assess the sustainability of the intervention and its effects. See Table 4 for an overview of data collection outcome measures, methods, participants and timelines.

**Table 4:** Data collection outcome measures, methods, participants and timelines of evaluation study

**Outcome measures**	**Data collection method**	**Participants and timelines**
**Patient clinical outcome measures**		
Demographic and clinical information (age, sex, duration of diabetes, smoking status, comorbidity)	Patient chart data extraction	20 different randomly-selected patient charts per site, per time interval (0, 6, 12, 18 and 24 months of intervention)
Hemoglobin A1c (HbA1c)		
Low-density lipoprotein (LDL-C)		
Total cholesterol-high density lipoprotein ratio (TC-HDL ratio)		
High density lipoprotein (HDL-C)				
Diastolic blood pressure (DBP)				
Systolic blood pressure (SBP)				
Glomerular filtration rate (eGFR)				
Albumin/creatinine ratio (ACR)				
Waist circumference				
Weight				
Body mass index (BMI)				
Treatment modality				
**Primary care physician (PCP) patient care process measures**		
HbA1c, blood pressure, lipid profile, nephropathy screening (ACR and eGFR) and foot exams/tests	Patient chart data extraction	20 different randomly-selected patient charts per site, per time interval (0, 6, 12, 18 and 24 months of intervention)
Referrals for dilated retinal exam		
Provisions for or recommendations of flu vaccine		
Changes to medication		
Differences in descriptive information across PCPs	PCP questionnaire on descriptive information (that is, sex, age, years practicing, type of practice, number of diabetes patients seen per month)	All PCPs, once
Patients’ experiences and views regarding Mobile Diabetes Education Team (MDET) intervention effectiveness	Patient in-depth interviews	20 randomly-selected patients at 12 months of intervention across sites
Scheduled and attended MDET appointments	Diabetes education program charts/forms	All MDET patients, up to 2 years of intervention
**Diabetes education program (DEP) utilization outcome measures**		
PCP referrals to DEPs	Diabetes education program charts	All patients, 12 months prior to start of intervention to 12 months following start of intervention
Patients’ utilization of DEPs		
**Inter-professional collaboration (IPC) and implementation process outcome measures**		
Collaborative Practice Assessment Tool (CPAT) scores of PCPs and MDET educators	CPAT	All PCPs at 12 months of intervention
		All educators at 12 months of intervention
PCPs’ experiences and views regarding the MDET intervention implementation and IPC	PCP in-depth interviews	16 randomly-selected PCPs at 12 months of intervention across sites
MDET educators’ experiences and views regarding the MDET intervention implementation and IPC	Educator in-depth interviews	16 randomly-selected educators at 12 months of intervention across sites
	Educator reflective journals	All educators, monthly, up to 2 years of intervention
	MDET debriefing sessions	All educators, quarterly, up to 2 years of intervention

### Quantitative data collection

A repeated cross-sectional survey of patients’ medical charts will be carried out at all sites at five time intervals (0, 6, 12, 18 and 24 months). This survey entails randomly selecting charts of eligible patients who received primary care at the participating site at the time of the survey. This results in different patients being included at the five time points. Therefore, data on the demographic and clinical profile of patients will be obtained and controlled statistically prior to performing outcome comparisons between study groups. In the control sites, a 12-month retrospective data extraction will also be performed for each patient chart selected. In the intervention sites, a 12-month retrospective data extraction will be performed prior to the start of the intervention and up to 12 months during the intervention for each patient chart selected. This will be conducted to assess changes in patient care processes and clinical outcomes.

Data will be collected on:

1. Baseline demographic and clinical information (that is, age, sex, duration of diabetes, smoking status, comorbidity);

2. Treatment modality (that is, diet, oral agents, insulin, or insulin with oral agents), medical treatment, duration of insulin therapy;

3. Frequency and outcomes of tests or measures of HbA1c, LDL-C, TC-HDL; high density lipoprotein (HDL-C), diastolic and systolic blood pressure, glomerular filtration rate (eGFR), albumin/creatinine ratio (ACR), waist circumference, and weight.

The data will be extracted from patient charts for baseline comparisons between randomized clusters. For our primary hypothesis, we will collect HbA1c to assess the proportion of patients who achieve the recommended target for HbA1c (that is, HbA1C ≤7%) and assess change within patients in the intervention and between cluster groups. For our secondary hypothesis we will collect LDL-C, TC-HDL, and diastolic and systolic blood pressure to assess achievement of each of the recommended clinical targets (LDL-C <2.0 mmol/L, TC-HDL ratio < 4.0 and blood pressure <130/80 mmHg) and assess change within patients in the intervention and between cluster groups.

The proportion of patient care processes completed by PCPs over a one-year period (that is, the percentage of patients with tests for HbA1c, blood pressure, lipid profile, screening for nephropathy (ACR and eGFR), foot exams, referrals for dilated retinal exams, and provisions for or recommendations of the flu vaccine) according to clinical practice guidelines will also be collected from patient charts and compared across study groups. We also plan to document and descriptively examine changes to medication (that is, change in drug, drug dose, and addition of another drug) to assess intensification of medical management and whether the diabetes educators had an influence on PCP practices. Descriptive information (that is, sex, age, years practicing) will be collected for each PCP to further assess differences in patient care processes between study groups.

We will also calculate the percentage of patient referrals by PCPs to hosting DEPs and the onsite MDET and compare this to patients’ actual attendance at DEPs and scheduled MDET appointments. The referral rate to DEPs will be compared to the percentage of referrals from participating PCPs and their patients’ attendance at DEPs a year prior to the start of the intervention. Characteristics available in patient medical charts (that is, sex, age, duration of diabetes, clinical information and so on) will be reviewed and compared between those who were and were not referred to the MDET to better understand PCP referral practices. The number of MDET appointments planned for each patient and the number of MDET visits attended will be collected during the intervention period to examine uptake of the MDET intervention.

Various characteristics of team collaboration will be measured using the Collaborative Practice Assessment Tool (CPAT) Version 2 [27] (Office of Interprofessional Education and Practice, Queen’s University, Kingston, Ontario Canada). The CPAT survey includes 56 items across 9 domains, including mission and goals; relationships; leadership; roles, responsibilities, and autonomy; communication; decision making; conflict resolution; community linkages and coordination; and perceived effectiveness and patient involvement, which are scored 1 (strongly disagree) to 7 (strongly agree), in addition to 3 open-ended questions. Individual scores will be aggregated to create an understanding of the overall team functioning at each site. The tool will be administered to PCPs and MDET educators after 12 months of the intervention.

### Qualitative data collection

Qualitative process data will be collected to describe how the intervention unfolds in practice and to assess the reliability of implementing the intervention across sites. The MDETs will be asked to record field notes of their experiences with the intervention, including their collaborations with PCPs, in a reflective journal. Quarterly debriefing sessions with the MDETs to discuss implementation issues that arise during the intervention period will be recorded, transcribed and analyzed for emergent themes, such as communication, shared decision making and so on.

In addition, purposively selected patients [44], MDET educators and PCPs will be asked to participate in a 45- to 60-minute open-ended qualitative face-to-face or telephone interview after 12 months from the start of the intervention. Potential participants will be stratified by sites, then invited to participate in an interview in order to ensure representation from all sites involved in the trial (maximum variation sampling) [45]. To obtain saturation for a heterogeneous group, interviews are planned with 15 to 20 people representing each group of patients, PCPs and educators who have participated in the MDET intervention. We will interview participants until saturation has been achieved (that is, when no new themes are being generated). Patient interview questions will pertain to how useful the intervention was in terms of better understanding their condition, diabetes self-management strategies, and the convenience and quality of delivery. Both MDETs and PCPs will be asked questions about their experiences with the intervention, their collaboration and teamwork, barriers and facilitators to implementation, and suggestions to improve the intervention. Participants will be compensated for their time. All interview questions will be piloted on two patients, PCPs and diabetes educators prior to interviews.

### Statistical analysis

All sites will eventually receive the MDET intervention on an ongoing basis following the stepped wedge design, which allows us to evaluate the intervention effect utilizing both within- and between-site information while controlling for underlying time trends (for example, Hussey *et al.*, 2007) [46]. We plan to sample 20 unique patients/charts per site per time period (1,200 in total), based on the primary hypothesis of a greater proportion of patients reaching the clinically recommended target (HbA1C ≤7%). The proposed sample size will give rise to over 80% power at a significance level of 1% (adjusting for multiple testing) assuming the between-site variation in terms of the coefficient of variation is 0.8. The minimal detectable effect size (expressed as a relative risk) based on the proposed sample is as small as 18%, for baseline proportions “p” in the range of 0.3 to 0.6 (as reported in McCrate *et al.*, 2010 [47], Harris *et al.*, 2005 [7] and Borgermans *et al.*, 2009 [15]]. For example, considering a conservative baseline proportion of *P* = 0.5 and an intraclass correlation (correlation between patients from the same site) of 0.39, we will have 80% power to detect a relative change in proportion of 18% (that is, from 0.5 to 0.59 ((0.59 to 0.5)/0.5 = 0.09/0.5 = 0.18)) attributed to the intervention over a six-month period. We will assess the sample size at the end of the 12 months, and if necessary, the sample size estimation will be adjusted according to established methods described in Chen *et al.* 2004 [48].

### Quantitative data analysis

Specifically, for Objective #1, we will test the effectiveness of the MDET intervention on clinical outcomes (for example, HbA1C ≤ 7%, LDL-C <2.0 mmol/L) using the generalized linear mixed models (GLMM), which include site as the random effect, time as a fixed effect and the intervention effect. With the GLMM approach, the temporal trends in the effect of the intervention are also evaluated, while the correlations between patients within the same site as well as the variations between sites are accounted for (Hussey *et al.*, 2007) [46]. The patient-level covariates (for example, age, sex, duration living with diabetes, smoking status) and PCP-level (within sites) covariates (for example, years of practice) will also be included in the model. Hierarchical linear models (HLM) [49,50] are an important special case of GLMM, and the former name is more commonly seen in the literature for nested data structures (for example, patients at Level 1, PCPs at Level 2 and sites at Level 3). GLMM will facilitate modeling all three level variances in patient outcomes, while utilizing patient variables at Level 1, PCP variables at Level 2 and site variables at Level 3. In addition, we will consider the generalized estimating equations (GEE) approach, which has been suggested to be more robust to misspecification of the variance structure (including. correlations between patients within sites) (Hussey *et al.*, 2007) [46]. For Objective #2, we will evaluate the effect of the MDET intervention on quality of care delivered to patients (for example, screening for ACR and eGFR) using the GLMM approach, as described for Objective #1, in addition to descriptive summaries (for example, for change in drug dose, PCP years practicing). GLMM will also be used to assess the intervention effect on, for example, the referral rate to DEPs under Objective #3, where the PCPs form the unit of the analyses, and the correlation between PCPs within sites will be taken into account along with the temporal trends of the MDET effects.

For the analyses described above, overall Type I error will be set to 1% (instead of 5%) for all calculations of confidence intervals and for testing the various hypotheses, in order to account for the possible occurrence of false positives due to “multiple testing” (increased chance of finding statistical significance beyond the nominal significance level). Model diagnostic tools and cross-validation methods will be considered for model assessment. Since missing values are unavoidable, these values will be handled with extra caution. Reasons and mechanisms for the missing data points will be explored by various summary statistics as well as graphical displays. Appropriate statistical techniques to handle missing values will be further explored and taken into account in the proposed statistical modeling [51], (including imputation of missing data). Statistical analyses will be carried out using the statistical software packages SAS (V9.2) (SAS Institute Inc., Cary, North Carolina, USA), and R (V2.12) (GNU Operating System, open source).

### Qualitative data analysis

All qualitative data collected will be analyzed by thematic analysis using a reciprocal coding approach [45,52], where researchers engage in open dialogue about themes and data interpretation. The theoretical framework underpinning the qualitative analysis is constructivist insofar as interpretation will take into account the multiplicities of views informing participants’ experiences [53]. Transcripts (that is, from field notes, debriefing sessions and interviews) will be first reviewed independently, and then through dialogue, and composite themes will be developed by at least two researchers. Thematic analysis, or pattern coding, is a method for grouping diverse sections of data into smaller analytic units [54]. A coding framework will be developed. MaxQDA Plus software (VERBI GmbH, Charlottenburg, Berlin, Germany) will be used to manage data and perform analysis. Preliminary findings will be shared with healthcare professionals to support the rigor and authenticity of the study findings.

## Discussion

We assume that the increasing prevalence of diabetes, combined with its complexity, rapidly evolving medical therapies and the requirement for patient self-management will lead to a growing demand for shared care across disciplines that target improvements in diabetes care and management [55]. We believe this model of care has the potential to improve professional practice and patient outcomes in diabetes care in our trial but also across Canada. In addition to strengthening and formalizing links between PCPs and DEPs, we expect that MDETs in primary care can increase timely access to DSMS, training and support, and improve patient experience and clinical outcomes through enhanced coordination and integration of care. The creation of inter-professional teams in primary care will provide a knowledge exchange opportunity by mentoring and developing diabetes proficiency in PCPs to improve knowledge and confidence in managing patients with diabetes and to refer more complex patients to specialized care.

This study is timely and relevant as DEPs and local PCPs are starting to integrate services across Canada to provide more team-based care to rectify the existing health service gaps demonstrated in the Canadian literature; thus, our research will provide health service policy makers with the necessary evidence to inform the practice of such a model in primary care. If MDETs are found to be feasible and effective, this alternative primary care model can be extended to other diabetes-related populations, such as those with impaired glucose tolerance and gestational diabetes/post-gestational diabetes. This MDET intervention can direct how diabetes prevention, management and care are delivered within primary care practice, and greatly reduce the burden of diabetes.

## Abbreviations

ACR: Albumin/creatinine ratio; CCM: Chronic care model; CPAT: Collaborative practice assessment tool; DEP: Diabetes education program; DSMS: Diabetes self-management support; DSMT: Diabetes self-management training; eGFR: Glomerular filtration rate; GEE: Generalized estimating equations; GLMM: Generalized linear mixed models; HbA1c: Hemoglobin A1c; HLM: Hierarchical linear models; IPC: Inter-professional collaboration; LDL-C: low-density lipoprotein; MDET: Mobile diabetes education team; PCP: Primary care physician; RD: Registered dietitian; RN: Registered nurse; SBGM: Self blood glucose monitoring; TC-HDL ratio: Total cholesterol-high density lipoprotein ratio.

## Competing interests

The authors declare that they have no competing interests.

## Authors’ contributions

EG and SH conceived of the study. EG, SH, MF, SE, WL, BS, SS and FW participated in the study design. EG and MF drafted the original research proposal. All authors read and provided feedback on the original proposal and approved the final manuscript.
